# First case of yellow fever in French Guiana since 1902.

**DOI:** 10.3201/eid0503.990314

**Published:** 1999

**Authors:** J M Heraud, D Hommel, A Hulin, V Deubel, J D Poveda, J L Sarthou, A Talarmin

**Affiliations:** Institut Pasteur de la Guyane, Cayenne, French Guiana.

## Abstract

The first case of yellow fever in French Guiana since 1902 was reported in March 1998. The yellow fever virus genome was detected in postmortem liver biopsies by seminested polymerase chain reaction. Sequence analysis showed that this strain was most closely related to strains from Brazil and Ecuador.


### First Case of Yellow Fever in French Guiana since 1902

**J.M. Heraud,* D. Hommel,† A. Hulin,† V. Deubel,‡ J.D. Poveda,‡ J.L. Sarthou,* and A. Talarmin***
*Institut Pasteur de la Guyane, Cayenne, French Guiana;†General Hospital, Cayenne, French Guiana; and ‡Institut Pasteur, Paris, France

The first case of yellow fever in French Guiana since 1902 was reported in March 1998. The yellow fever virus genome was detected in postmortem liver biopsies by seminested polymerase chain reaction. Sequence analysis showed that this strain was most closely related to strains from Brazil and Ecuador.

Yellow fever (YF) is a serious public health problem in many tropical countries in Africa and South America. In South America, most infections are sporadic, affecting unvaccinated persons who enter the forest; monkeys are the primary reservoirs, and *Haemagoggus* sp. are the vectors. French Guiana, located between Brazil and Surinam in the Amazonian forest, has had many epidemics of YF. However, no case has been reported in French Guiana since 1902, although a serologic survey in 1951 found circulating virus; of 430 persons younger than 50 years of age (who had not been affected by the 1902 epidemic), 9% of those living in the coastal area and 29% of those living inland had significant titers of neutralizing YF antibodies (1). A study conducted at the same time in Surinam showed even higher rates of YF seropositivity in persons who had not been exposed to previous epidemics and confirmed that YF viruses were circulating in the region (2). In French Guiana, YF immunization became compulsory in 1967.

### Case Report

In March 1998, an Amerindian woman living in a forest area on the Maroni River was admitted to the health center in Maripasoula, French Guiana, with fever, headache, abdominal pain, vomiting, and diarrhea. She was treated for malaria because of a *Plasmodium falciparum*–positive blood smear. Two days later, the patient's fever increased (40.2°C), she became jaundiced, and she was evacuated to the intensive care unit (ICU) at Cayenne Hospital with multiple visceral failure: shock syndrome, renal failure (blood urea level 32 mmol/l, creatinine level 656 µmol/l), and liver failure (total bilirubin level 314 µmol/l, alanine aminotransferase 2048 IU/l, aspartate amino transferase 6256 IU/l, prothrombin level 23%). No hemorrhages were noted. Despite symptomatic treatment, the condition of the patient deteriorated rapidly, and she died a few hours after admission to ICU.

Blood cultures were negative for bacterial pathogens. Because of anuria, urine cultures were not possible, and albuminuria could not be tested. Examination of peripheral blood smears showed no parasites on admission to ICU and titers of antibodies to leptospira were low.

Microscopy examination of postmortem liver biopsies showed histopathologic changes characteristic of YF: midzonal necrosis with a small rim of a few viable periportal and pericentral hepatocytes and centrilobular cells with microsteatosis and eosinophilic degeneration with round, eosinophilic cytoplasmic structures (Councilman bodies).

A serum sample collected before death and a serum sample obtained from the patient in 1994 during a seroepidemiologic study on HTLV-I infection and stored at -80°C at the Institut Pasteur de la Guyane, French Guiana, were tested for immunoglobulin (Ig) G and IgM specific for three flaviviruses (YF, dengue, and Saint Louis encephalitis), two alphaviruses Tonate and Mayaro), and a new world

Address for correspondence: A. Talarmin, Institut Pasteur de la Guyane, 23 Avenue Pasteur, BP 6010, 97306 Cayenne Cedex, Guyane Française; fax: 0594-309-416; e-mail: atalarmin@pasteur-cayenne.fr.

arenavirus (Tacaribe), by enzyme-linked immunosorbent assay (3). A plaque reduction neutralization test was also used to detect YF-neutralizing antibodies (4). The serum collected in 1998 contained IgM (but not IgG) that specifically recognized YF antigens. IgM specific for other flavivirus antigens (dengue, Saint Louis encephalitis) were not found. The neutralizing antibody titer of the 1998 serum as assessed by plaque reduction neutralization test was 20. In the serum collected in 1994, no YF virus–specific antibodies were detected by any technique. IgG to Mayaro virus was detected in both sera; no other antibodies to alphaviruses and arenaviruses were detected.

Serum and homogenized liver samples from the patient were diluted 10-fold in Leibowitz medium containing 3% fetal calf serum, and dilutions were injected into subconfluent AP 61 cell cultures (5). After 7 days, cells were harvested and tested for YF virus by an indirect immunofluorescence assay using a monoclonal antibody from the Centers for Disease Control and Prevention, Fort Collins, CO, USA. YF virus was not isolated by cell culture from either blood or liver samples.

Reverse transcription–polymerase chain reaction (RT-PCR) tests were performed on RNA extracted from serum and liver (6). RNA of the YF virus was detected by RT-PCR after seminested PCR only in the liver sample. The 542-bp PCR product was purified and directly sequenced with an automatic sequencing system (ACTgene, EuroSequence Gene Services, Evry, France). The first 309 nucleotides of the 3' noncoding sequence were aligned with those of sequenced YF strains from Genbank (7,8). Sequences were aligned with the Clustal W program. Bootstrap confidence limits were calculated from 100 replicates with the program SEQBOOT. Phylogenetic analyses were performed by maximum parsimony by using the DNAPARS program with uniform character weights and a heuristic search option. All branch lengths were drawn to scale by the program Treetool. The sequence of the YF strain isolated in 1998 was deposited in Genbank (accession number: AF121952).

The sequence of the amplified gene differed at 11 positions (3.5% nucleotide divergence) from that of a Brazilian strain isolated in 1935 and at 21 positions (6.8% nucleotide divergence) from that of a strain isolated in Ecuador in 1979. The sequence diverged more from strains isolated in Peru (1995) and Trinidad (1979) (8.1% and 10% divergence, respectively). As expected, African strains differed more, with those of West Africa (from Nigeria and Senegal) being less distant than those from East and Central Africa (Uganda and Central African Republic). The nucleotide sequence downstream from the NS5 stop codon in the 3' noncoding region of the YF virus was deleted in the French Guianese strain, as in several South American YF viruses (9). Phylogenetic analysis of the sequences confirmed that the virus was most closely related to those isolated in Brazil and Ecuador (Figure).

### Conclusions

The histopathologic changes of the liver were characteristic of YF but also of other hemorrhagic fever viruses. The IgMs were only slightly above the cut-off values used in the laboratory. Although YF was highly probable, the diagnosis required confirmation by detection of the virus or its genome. Indeed, in 1990 a suspected case of YF was reported to the World Health Organization because of characteristic histopathologic changes, but the case was never confirmed (presence of IgG but no IgM specific for YF, no detection of the virus in liver or serum); later it was shown that the patient had been vaccinated against YF 1 year before (10). However, because YF requires health authorities to take specific measures, confirmation of the diagnosis is important, especially when YF is not prevalent. The case we described was confirmed by RT-PCR from liver only, because the serum sample was taken on day 6 when viremia is usually resolved and because the sensitivity of cell culture for virus in liver samples is very low (probably because of biliary salts toxicity). This case underscores the need for postmortem liver biopsies for detecting the viral genome to confirm diagnosis.

This patient did not leave her village the months before infection; she was probably infected while working in forest clearings. The detection of a YF virus in French Guiana nearly a century after the last report is notable; however, the absence of reported cases during the previous years is surprising because no natural borders exist between this country and northern Brazil, where YF is not uncommon (11). A severe YF outbreak would have easily been detected, but sporadic cases can be misdiagnosed

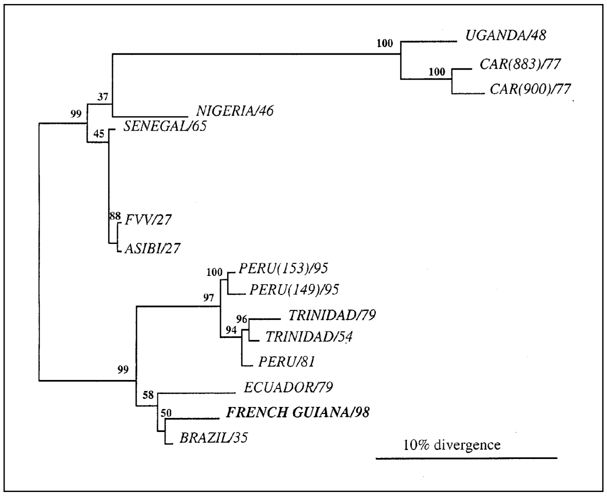

Figure. Phylogenetic tree generated from 309 nucleotides of the 3' noncoding region of the strain of yellow fever (YF) isolated in French Guiana in 1998 (in bold) and of 14 other YF strains by using the DNAPARS program. Numbers indicate bootstrap values for groups to the right. One µl (30 ng) of primer VD8 (5'-GGGTCTCCTCTAACCTCTAG-3') was mixed with RNA resuspended in 10 µl of distilled water; the mixture was heated at 95°C for 2 minutes and placed on ice. cDNA was synthesized in a mixture containing reverse transcriptase (RT) incubation buffer (provided by the manufacturer), 0.2 mM of each of the four dNTPs, 20 units RNasine, and five units of AMV RT (Promega, Charbonnières, France) by incubation for 1 hour at 42°C. cDNA was amplified by PCR. Four µl of the cDNA sample was added to 46 µl of a mixture containing Taq polymerase buffer (provided by the manufacturer), 2 mM $MgCl_2$, 0.5 mM of each of the 4 dNTPs, 300 ng of primer VD8 and of degenerate primer EMF1 (5'-TGGATGACSACKGARGAYATG-3') (S = C, G; K = G, T; R = A, G; Y = C, T), and 0.5 unit of Taq polymerase (Promega, Charbonnières, France). After 5 minutes of denaturation at 95°C, the mixture was subjected to 30 polymerase chain reaction (PCR) cycles: 95°C for 30 seconds, 53°C for 90 seconds, and 72°C for 60 seconds, followed by a final 10-minute polymerization step at 72°C. Four µl of a 1 in 100 dilution of the PCR products was used for seminested PCR using primers VD8 and NS5YF (5'-ATGCAGGACAAGACAATGGT-3'). After the denaturation step, DNA was amplified by 25 cycles of PCR: 94°C for 30 seconds, 55°C for 90 seconds, and 72°C for 120 seconds, followed by a final extension step at 72°C. Negative controls (serum from healthy persons) were included in the series. The positive control (supernatant of infected mosquito cells) was tested separately to avoid any contamination. The phylogenetic analysis was conducted by the Pasteur Institute in Paris.

as other fevers or as hepatitis (when jaundice is present) and may be not tested for YF even though serologic and YF virus detection tests are performed for each suspected case.

No other case was diagnosed in the patient's family or neighborhood, but sporadic cases are common in South America (12), probably because of poorly anthropophilic vectors. Furthermore, in this area, approximately 90% of the population have been vaccinated at least once (R. Pignoux, unpub. data). Outbreaks are common among nonhuman primates, but no epidemic has occurred in the area where the patient lived. However, YF incidence increased in northern Brazil in 1998 (13).

This case calls attention to vaccination problems in French Guiana, especially along the rivers. Our patient had been vaccinated in 1985, but the absence of neutralizing antibodies in 1994 indicates that the vaccination was not effective. Although this patient may have had a poor antibody response, more likely inadequate storage of the vaccine before use was responsible. In 1985, YF vaccines were less thermostable than now, and the cold chain was difficult to maintain. In response to this case, an immunization campaign was initiated in this area in May.

Vaccination of the population must continue since YF can reappear. The immunization program implemented in French Guiana (compulsory vaccination of children older than 1 year of age, booster YF vaccination every 10 years, and required vaccination certificate before entering school) should avert the threat of outbreaks in urban areas, which have a vaccine coverage rate of approximately 80%. However, the risk for sporadic cases in unvaccinated persons will persist, and so active serologic and virologic surveillance of YF remains necessary.

### Acknowledgment

The authors thank Michel Favre for his help in the phylogenetic analysis of the yellow fever virus sequences.

This study was supported by a grant from the Programme Hospitalier de Recherche Clinique.

Jean-Michel Heraud is a Ph.D. student working in the virology laboratory of the Pasteur Institute of French Guiana. His areas of expertise are virology and hematology with a focus on arbovirus epidemiology and physiopathology.

### References

1. Floch H, Durieux C, Koerber R. Enquête épidémiologique sur la fièvre jaune en Guyane française. Annales Institut Pasteur 1953;84:495-508.
2. Wolff JW, Collier WA, De Roever-Bonnet H, Hoekstra J. Yellow fever immunity in rural population groups of Surinam. Tropical and Geographical Medicine 1958;325-31.
3. Lhuillier M, Sarthou JL. Intérêt des IgM anti-amariles dans le diagnostic et la surveillance épidémiologique de la fièvre jaune. Annales de Virologie (Institut Pasteur) 1983;134E:349-59.
4. Lindsey HS, Calisher CH, Matthews JH. Serum dilution neutralization test for California group virus identification and serology. J Clin Microbiol 1976;4:503-10.
5. Reynes JM, Laurent A, Deubel V, Telliam E, Moreau JP. The first epidemic of dengue hemorrhagic fever in French Guiana. Am J Trop Med Hyg 1994;51:545-53.
6. Deubel V, Huerre M, Cathomas G, Drouet M-T, Wuscher N, Le Guenno B, et al. Molecular detection and characterization of yellow fever in blood and liver specimens of a non-vaccinated fatal human case. J Med Virol 1997;53:212-7.
7. Hahn CS, Dalrymphe JH, Strauss JH, Rice CM. Comparison of the virulent Asibi strain of yellow fever virus with the 17D vaccine strain derived from it. Proc Natl Acad Sci U S A 1987;84:2019-23.
8. Wang E, Ryman KD, Jenings AD, Wood DJ, Taffs F, Minor PD, et al. Comparison of the genomes of the wild-type French viscerotropic strain of yellow fever virus with its vaccine derivative French neurotropic vaccine. J Gen Virol 1995;76:2749-55.
9. Wang E, Weaver SC, Shope RE, Tesh RB, Watts DM, Barett ADT. Genetic variation in yellow fever virus: duplication in the 3' noncoding region of strains from Africa. Virology 1996;225:274-81.
10. World Health Organization. Yellow fever in 1989 and 1990. Wkly Epidemiol Rec 1992;67:245-51.
11. World Health Organization. Yellow fever in 1994 and 1995. Wkly Epidemiol Rec 1996;71:313-8.
12. Tolou H. La fièvre jaune: aspects modernes d'une maladie ancienne. Méd Trop 1996;56:327-32.
13. World Health Organization. Yellow fever in Brazil. Wkly Epidemiol Rec 1998;7:351.

